# Effects of fluid flow shear stress to mouse muscle cells on the bone actions of muscle cell-derived extracellular vesicless

**DOI:** 10.1371/journal.pone.0250741

**Published:** 2021-05-07

**Authors:** Yoshimasa Takafuji, Kohei Tatsumi, Naoyuki Kawao, Kiyotaka Okada, Masafumi Muratani, Hiroshi Kaji

**Affiliations:** 1 Department of Physiology and Regenerative Medicine, Kindai University Faculty of Medicine, Osaka, Japan; 2 Faculty of Medicine, Department of Genome Biology, University of Tsukuba, Tsukuba, Japan; Mayo Clinic Minnesota, UNITED STATES

## Abstract

The interactions between skeletal muscle and bone have been recently noted, and muscle-derived humoral factors related to bone metabolism play crucial roles in the muscle/bone relationships. We previously reported that extracellular vesicles from mouse muscle C2C12 cells (Myo-EVs) suppress osteoclast formation in mice. Although mechanical stress is included in extrinsic factors which are important for both muscle and bone, the detailed roles of mechanical stress in the muscle/bone interactions have still remained unknown. In present study, we examined the effects of fluid flow shear stress (FFSS) to C2C12 cells on the physiological actions of muscle cell-derived EV. Applying FFSS to C2C12 cells significantly enhanced muscle cell-derived EV-suppressed osteoclast formation and several osteoclast-related gene levels in mouse bone marrow cells in the presence of receptor activator nuclear factor κB ligand (RANKL). Moreover, FFSS to C2C12 cells significantly enhanced muscle cell-derived EV-suppressed mitochondria biogenesis genes during osteoclast formation with RANKL treatment. In addition, FFSS to C2C12 cells significantly enhanced muscle cell-derived EV-suppressed osteoclast formation and several osteoclast-related gene levels in Raw264.7 cells in the presence of RANKL. Small RNA-seq-analysis showed that FFSS elevated the expression of miR196a-5p and miR155-5p with the suppressive actions of osteoclast formation and low expression in mouse bone cells. On the other hand, muscle cell-derived EVs with or without FFSS to C2C12 cells did not affect the expression of osteogenic genes, alkaline phosphatase activity and mineralization in mouse osteoblasts. In conclusion, we first showed that FFSS to C2C12 cells enhances the suppressive effects of muscle cell-derived EVs on osteoclast formation in mouse cells. Muscle cell-derived EVs might be partly involved in the effects of mechanical stress on the muscle/bone relationships.

## Introduction

Numerous clinical studies about the relationships between sarcopenia and osteoporosis suggest that skeletal muscle and bone interact each other [1,2]. Among the muscle/bone interactions, it has been recently recognized that muscle-secreted factors, myokines, such as myostatin, irisin, follistatin, interleukin-6, insulin-like growth factor (IGF)-1, fibroblast growth factor (FGF)-2 and osteoglycin, regulate bone metabolism [3–6].

Mechanical stress is the critical extrinsic factor for both muscle and bone. Mechanical unloading, which occurs in bedridden or microgravity during space flight, induces muscle atrophy and bone loss in humans and animals [3]. As for the effects of mechanical stress on the muscle/bone relationships, we previously showed that irisin is involved in mechanical unloading-induced muscle wasting and osteopenia as a myokine linking muscle to bone in mice [5]. We revealed that follistatin, a physiological myostatin inhibitor, as well as olfactomedin1 are involved in muscle and bone alterations induced by gravity change in mice [4,7]. Moreover, we showed that muscle and serum level of Dickkopf-related protein 2, an inhibitor of Wnt/β-catenin signaling, was positively and negatively regulated by mechanical unloading and hypergravity in mice [8]. On the other hand, several studies suggested the importance of osteocyte-derived sclerostin in the effects of mechanical stress in the linkage of bone to muscle [9,10], although sclerostin is well known humoral factor secreted from osteocytes regulated by mechanical stress [11]. Moreover, Juffer et al reported that mechanical loading increases the expression of IGF-I, vascular endothelial growth factor and hepatocyte growth factor in osteocytic MLO-Y4 cells [12]. Taken together, mechanical stress might affect the muscle and bone interactions via regulating the production of humoral factors.

Extracellular vesicles (EVs) containing various microRNAs (miRNAs) or proteins are secreted from various tissue and circulate in body fluids. EVs play a crucial role in physiological and pathophysiological processes by transferring their contents to distant tissues [13]. Qin et al reported that myostatin inhibits differentiation of osteoblasts through the suppression of miR-218 in exosomes secreted from osteocyte [14]. We recently reported that EVs secreted from C2C12 cells (Myo-EVs) suppress osteoclast formation and mitochondrial energy metabolism in mouse bone marrow and Raw264.7 cells [12], and miR-196a-5p included in Myo-EVs might be responsible for the effects of Myo-EVs on osteoclast formation [15,16]. These findings suggest that EVs might be a crucial mediator in the muscle/bone interactions. However, the roles of muscle-derived EVs in the muscle bone interactions in the physiological and pathophysiological states have still remained unknown.

Recent studies showed that mechanical stress to osteocytes increases secretion of osteocyte-secreting EVs and expression of miR181b-5p in osteocyte-secreting EVs, which contribute to osteogenic differentiation and bone formation [17,18]. Moreover, Hua et al reported that mechanical stretch regulates the miRNA expression profiles in C2C12 cells via NF‑κB activation [19]. Taken together, mechanical stress might modulate the effects of EVs on the muscle/bone interaction. However, the effects of mechanical stress on muscle-derived EVs in the muscle/bone interactions have not been reported so far to our knowledge. We therefore examined the effects of mechanical stress to mouse muscle C2C12 cells on the physiological actions of muscle cell-derived EVs on mouse bone cells.

## Materials and methods

### Isolation of EVs from C2C12 cells

Mouse muscle C2C12 cells (obtained from American Type Culture Collection (ATCC), Manassas, VA) were cultured in high-glucose Dulbecco’s modified Eagle’s medium (DMEM; Wako, Osaka, Japan) supplemented with 10% fetal bovine serum that was heat inactivated in 56°C for 30min (FBS; GE Healthcare Japan, Tokyo, Japan) and 1% penicillin/streptomycin (PS). Six-well plate and 10 cm culture plate was coated with collagen (Cellmatrix type I-C, Nitta Gelatin, Inc., Osaka, Japan). C2C12 cells were seeded on the plates in same density (1×10^5^ cells/cm^2^) and cultured at 37 ^ο^C for 24 hr. EVs contained in FBS for culturing C2C12 cells were depleted by ultracentrifugation at 130,000 g for 16 hr at 4°C in advance.

Applying fluid flow shear stress (FFSS) is an useful method for loading mechanical stress to cultured cells [4,20], and the maximal FFSS at the bottom of the plate can be calculated from following equation, FFSS (dyn/cm^2^) = α √[ρη(2πf)^3^[α: the radius of gyration of the shaker (cm); ρ: the density of the medium (g/mL); η: the dynamic viscosity (7.5 × 10^−3^ dyn/cm^2^, at 37°C); f: the frequency of rotation (rotations per second)]. Confluent C2C12 cells were replaced with fresh DMEM supplemented with 10% EV-depleted FBS. For analysis of the effects of shear stress on physiological action of Myo-EV, C2C12 cells cultured in 6-well plates were placed on an orbital shaker (radius of gyration is 0.5 cm, KS 260 basic; IKA, Osaka, Japan) that was set to rotate at 250 rpm (calculated shear stress is 6 dyn/cm^2^) in an incubator and cultured for 48 hr at 37°C.

EVs were isolated from conditioned medium (CM) of C2C12 cells by ultracentrifugation as described previously [15,16,21]. Briefly, the CM was centrifuged (3,000 g, 5 min, 4^ο^C) for removing dead cells and filtered with a 0.22-μm PVDF filter (Millipore, Bedford, MA, USA) for removing cell debris. Then, the CM was ultracentrifuged (130,000 g, 70 min, 4 ^ο^C) with a Himac CP80NX system (HITACHI, Tokyo, Japan) for isolating pellet of EVs. The pellet was suspended in fresh PBS (Myo-EVs), and protein amount of the Myo-EVs was measured with a BCA Protein Assay Kit (Pierce, Rockford, IL). The suspended Myo-EVs were stored at −80 ^ο^C.

Size distribution of the isolated Myo-EVs was measured with a NanoSight LM10V-HS system (Malvern Instruments, Malvern, UK) as described previously [16,21]. The quantitation was repeated five times within one measurement (the mean values were shown in figure).

### Animals

Male mice with a mixed C57BL/6J (81.25%) and 129/SvJ (18.75%) genetic background (provided by D. Collen (University of Leuven, Leuven, Belgium)) were bred and used for experiment. The mice fed a normal diet, and food and water were available as libitum. We performed all animal experiments at Kindai University Faculty of Medicine animal facility according to the guidelines of the National Institutes of Health and the institutional rules for the use and care of laboratory animals at Kindai University. All animal experiments were approved by the animal ethics committee of Kindai University (Number: KAME-31-051). All surgery was performed under anesthesia with excess isoflurane, and all efforts were made to minimize suffering.

### Osteoclast formation

Method for analysis of osteoclast formation was reported previously [15,16,22]. Briefly, we isolated bone marrow cells from the tibiae of one male mouse (8 week-old) after sacrificed by cervical dislocation under 2% isoflurane anesthesia. For osteoclast formation and quantitative real-time PCR analyses, the bone marrow cells were seeded in 96-well and 24-well plates (cell density: 1×10^5^ cells/cm^2^) respectively, and cultured in α-MEM (Wako) supplemented with 10% FBS, 1% PS and macrophage colony-stimulating factor (M-CSF, Wako) for 3 days at 37°C for forming bone marrow-derived macrophages (BMMs). For osteoclast formation, the BMMs were cultured in the presence of receptor activator of nuclear factor κB ligand (RANKL, Wako) and M-CSF for an additional 4–5 days at 37°C.

Mouse monocytic Raw264.7 cells (obtained from ATCC) were seeded in 96-well and 24-well plates (cell density: 1×10^4^ cells/cm^2^) for osteoclast formation and quantitative real-time PCR, respectively. The cells were cultured in α-MEM supplemented with 10% FBS, 1% PS and RANKL for 5 days at 37°C.

The cells were stained with a tartrate-resistant acid phosphatase (TRAP) staining kit (Wako). Number of TRAP-positive multinucleated cells (MNCs) containing three or more nuclei in each well of 96-well plates was counted as osteoclast.

### Quantitative real-time PCR

Quantitative real-time PCR was performed with the method similar with previous reports [15,16]. Total RNA of cells was extracted with the Nucleo Spin®RNA Plus (Takara Bio, Shiga, Japan). Total RNA of EVs was extracted with RNeasy Mini Kit (Qiagen, Hilden, Germany).

Total RNA (500 ng) was reverse transcribed into complementary DNA (cDNA) for mRNA quantification using the Prime Script RT reagent Kit with gDNA eraser (Takara Bio). A quantitative real-time PCR for mRNA was performed using SYBR Premix Ex Taq™ II kit (Takara Bio) and ABI Step One Plus Real-Time PCR System (Applied Biosystems, Foster, CA, USA). The mRNA levels of target genes were determined as the Ct value, which were normalized with GAPDH. Each primer sequence (forward and reverse) is shown in Table 1.

**Table 1:** Sequences of the primers for real-time PCR.

*CTSK*	Forward	5’-GTTACTCCAGTCAAGAACCAGG-3’
	Reverse	5’-TCTGCTGCACGTATTGGAAGG-3’
*CytC*	Forward	5’-GGAGGCAAGCATAAGACTGG-3’
	Reverse	5’-TCCATCAGGGTATCCTCTCC-3’
*DC-STAMP*	Forward	5’-TATCTGCTGTATCGGCTCAT-3’
	Reverse	5’-AGAATAATACTGAGAGGAACCCA-3’
*Gapdh*	Forward	5′-AGGTCGGTGTGAACGGATTTG-3′
	Reverse	5′-GGGGTCGTTGATGGCAACA-3′
*ND4*	Forward	5’-CATCACTCCTATTCTGCCTAGCAA-3’
	Reverse	5’-TCCTCGGGCCATGATTATAGTAC-3’
*NFATc1*	Forward	5’-GGAGAGTCCGAGAATCGAGAT-3’
	Reverse	5’-TTGCAGCTAGGAAGTACGTCT-3’
*OPG*	Forward	5′-AGTCCGTGAAGCAGGAGT-3′
	Reverse	5′-CCATCTGGACATTTTTTGCAAA-3′
*Osterix*	Forward	5’-AGCGACCACTTGAGCAAACAT-3’
	Reverse	5’-GCGGCTGATTGGCTTCTTCT-3’
*PGC1-β*	Forward	5’-CCTCATGCTGGCCTTGTCA-3’
	Reverse	5’-TGGCTTGTATGGAGGTGTGG-3’
*RANKL*	Forward	5′-CACAGCGCTTCTCAGGAGCT-3′
	Reverse	5′-CATCCAACCATGAGCCTTCC-3′
*RUNX2*	Forward	5’-AAATGCCTCCGCTGTTATGAA-3’
	Reverse	5’-GCTCCGGCCCACAAATCT-3’
*TRAP*	Forward	5’-GCAACATCCCCTGGTATGTG-3’
	Reverse	5’-GCAAACGGTAGTAAGGGCTG-3’

Total RNA (100 ng) was reverse transcribed into cDNA for miRNA quantification using TaqMan reverse transcription kit (Thermo Fisher Scientific, Inc., Waltham, MA, USA) and TaqMan miRNA assay kit (Thermo Fisher Scientific, Inc). A quantitative real-time PCR for miR155-5p (Assay ID: 002571), miR196a-5p (Assay ID: 241070) and small nucleolar RNA-202 (snoRNA202; Assay ID: 001232) was performed using the TaqMan MicroRNA assay kit and ABI Step One Plus Real-Time PCR System. The expression levels of miR196a-5p were determined as the Ct value and normalized with snoRNA202 as an endogenous control.

### Primary osteoblast isolation and culture

Primary osteoblasts was isolated from the calvariae of neonatal mice as reported previously [15,16,23]. Briefly, we isolated the calvariae from five male mice (3- to 5-day-old) under 2% isoflurane anesthesia and digested the calvariae with 1 mg/mL collagenase and 0.25% trypsin four times for 20 min at 37°C. Isolated cells from the second, third and fourth digestions were seeded in 6-well plates (cell density: 1×10 ^4^ cells/cm^2^) and cultured in α-MEM supplemented with 10% FBS and 1% PS until confluent. Osteoblasts at second passage were used for the experiments. The osteoblasts were cultured in the presence of 50 μg/mL ascorbic acid and 10 mM β-glycerophosphate for 2 weeks for the mineralization assay. The cells were stained with Alizarin red (Kishida Chemical, Osaka, Japan) and treated with ethylpyridinium chloride (Wako). The absorbance at 550 nm of the extracted stain was measured for evaluation of mineralization.

### Oxygen consumption measurement

Mouse bone marrow cells were analyzed with an XF96 Extracellular Flux Analyzer with a Mito Stress kit (Seahorse Bioscience, North Billerica, MA, USA) for measurement of the oxygen consumption rate (OCR). Mouse bone marrow cells were seeded (5×10^3^ cells/well) and cultured with 50 ng/mL M-CSF for 3 days in XF96 cell culture microplates. Then, the cells were cultured with 50 ng/mL RANKL and 50 ng/mL M-CSF for an additional 5 days. The XF96 analyzer measured the basal OCR and the OCR after injection of various inhibitors, oligomycin (1 μM), FCCP (0.5 μM) and rotenone (0.5 μM)/antimycin A (0.5 μM), for three measurement cycles at each step. Mitochondrial function, such as basal respiration, ATP production, maximal respiration and non-mitochondrial respiration, was determined as previously reported [15,16]. The time points for injection of various inhibitors were shown with arrows in figure.

### Small RNA sequencing (RNA-seq) analysis

For RNA-seq analysis, total RNA was extracted from Myo-EVs, primary mouse bone marrow cells and osteoblasts with TRIZOL reagent (Thermo Fisher Scientific, Inc) and assessed by an Agilent Bioanalyzer with the RNA 6000 Pico Kit (Agilent, Santa Clara, CA, USA). Sequencing library was prepared using the NEBNext Small RNA Library Prep Kit (E7330, New England Biolabs, Ipswich, MA, USA). Total RNA (90 ng) was ligated to 3’ Adaptor, and excess adaptor was subsequently absorbed. RNA fragments were further ligated to 5’ Adaptor for cDNA synthesis. Synthesized cDNA was amplified by PCR for 12 cycles. Small-RNA library was size-selected by AMPure beads (NC9933872, Thermo Fisher Scientific, Inc) and verified with the Bioanalyzer DNA High-sensitivity Kit (5067–4626, Agilent). For obtaining 2 × 36-base paired-end reads, sequencing was performed by the Illumina NextSeq500 (Illumina, San Diego, CA, USA). FASTQ files were imported to CLC Genomics Workbench (Ver.10.1.1, Qiagen, Germantown, MD, USA). Reads were grouped by sequence and the read groups were matched to miR-base annotated micro RNAs. Raw counts of each sample were converted by total count 1,000,000 at first, and the converted counts were further normalized by scaling method with means as normalization values, median mean as reference and trimming percentage 5 for comparing multiple samples.

### Statistical analysis

All data in the present study are shown as the mean ± the standard error of the mean (SEM). For multiple comparisons, one-way ANOVA followed by the Tukey-Kramer post hoc test was used. The Mann-Whitney U test was used to compare two groups. The significance level was set at p<0.05, and all statistical analyses were performed by GraphPad PRISM 6 software (La Jolla, CA).

## Results

### Analyses of EVs secreted from C2C12 cells exposed with or without fluid flow shear stress

**Fig 1:**
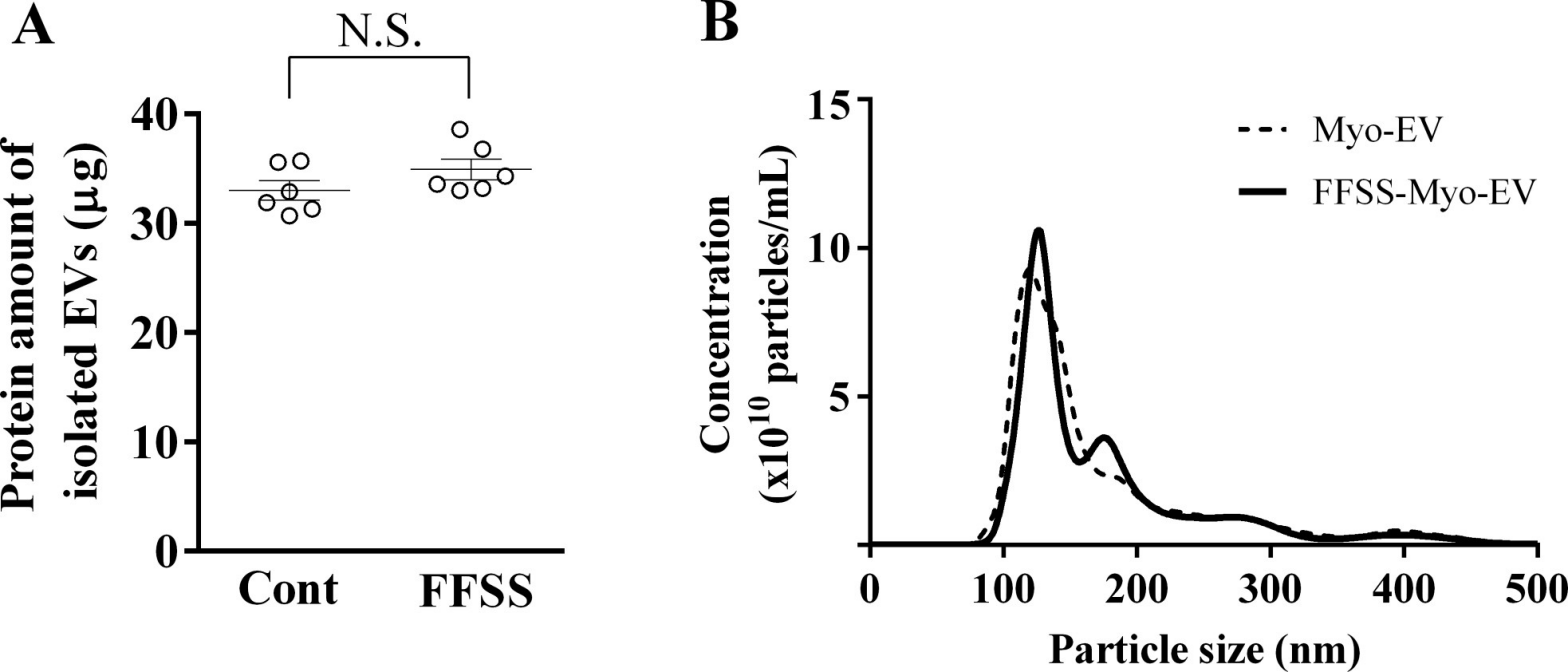
Analysis of isolated EVs from C2C12 cells (Myo-EVs). (A) The protein amount of Myo-EVs (Cont) secreted from C2C12 cells and Myo-EVs secreted from C2C12 cells exposed with FFSS (FFSS-Myo-EVs; FFSS) in 6-well plates were measured. Data represent mean ± SEM of 6 experiments in both groups. (B) The particle size and concentration of Myo-EVs and FFSS-Myo-EVs were measured with a NanoSight LM10V-HS system. Data represent mean of 5 determinations within one experiment in both groups. N.S: not significant.

Conditioned medium of mouse C2C12 cells exposed with or without FFSS was ultracentrifugated for collecting EVs. The protein amounts of Myo-EVs and EVs secreted from C2C12 cells exposed with FFSS (FFSS-Myo-EVs) were not significantly different (Fig 1A). The particle concentration and size distribution of Myo-EVs and FFSS-Myo-EVs were measured using a NanoSight LM10V-HS system. The particle sizes of Myo-EVs and FFSS-Myo-EVs were almost less than 200 nm (Fig 1B). This finding suggests that the collected Myo-EVs and FFSS-Myo-EVs are exosome-rich. In addition, size distribution of Myo-EVs and FFSS-Myo-EVs were similar (Fig 1B). These results suggest that exposing FFSS does not affect the amount and size of EVs secreted from C2C12 cells.

### Effects of FFSS to C2C12 cells on muscle cell-derived EV-suppressed osteoclast formation

**Fig 2:**
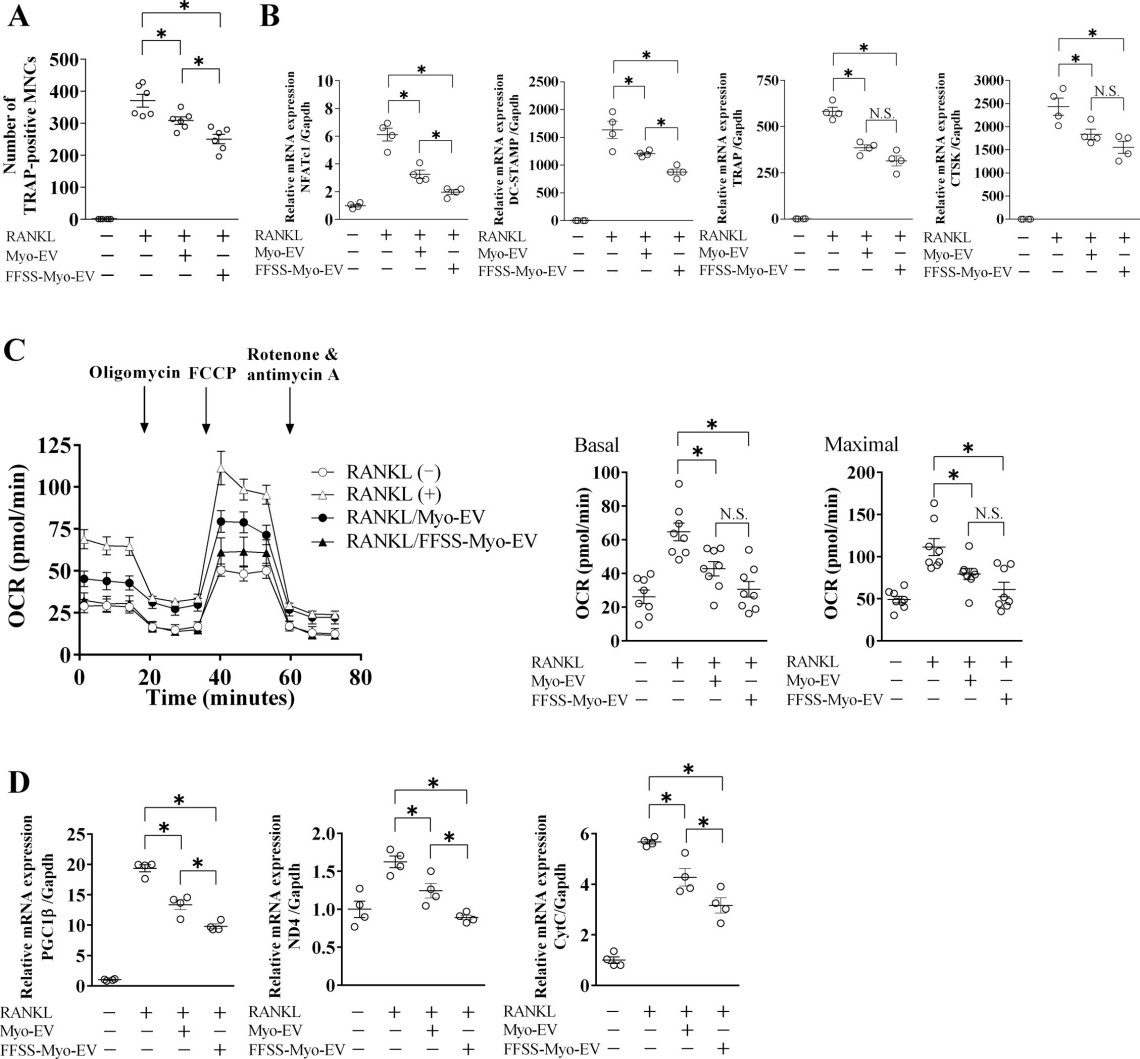
Effect of Myo-EVs secreted from C2C12 cells exposed with or without FFSS on osteoclast formation and mitochondrial biogenesis from mouse bone marrow cells. Osteoclast precursors were induced from bone marrow cells isolated from mice by culturing with M-CSF (50 ng/mL) for 3 days. (A) Osteoclast precursors were cultured with and without Myo-EVs or FFSS-Myo-EVs (1 μg/mL) in the presence or absence of M-CSF (50 ng/mL) and RANKL (50 ng/mL) for 5 days. Numbers of TRAP-positive multinucleated cells (MNCs) in a 96-well plate were quantified. Data represent mean ± SEM of 6 experiments in each group. (B) Osteoclast precursors were cultured with and without Myo-EVs or FFSS-Myo-EVs (4 μg/mL) in the presence or absence of M-CSF (50 ng/mL) and RANKL (50 ng/mL) for additional 4 days. Total RNA of cells was extracted for gene expression analysis of NFATc1, DC-STAMP, TRAP, CTSK or Gapdh by quantitative real-time PCR. Data represent mean ± SEM of 4 experiments in each group and are expressed relative to Gapdh mRNA values. (C) The oxygen consumption rate (OCR) of mouse bone marrow cells was analyzed with an XF96 Extracellular Flux Analyzer. Basal OCR (before stimulation with oligomycin) and maximal OCR (after stimulation with FCCP) were measured. Data represent mean ± SEM of 8 experiments in each group. (D) Gene expression levels of mitochondrial biogenesis markers [PGC1β, ND4 and Cytochrome C (CytC)] or Gapdh in bone marrow cells were analyzed. Data represent mean ± SEM of 4 experiments in each group, are expressed relative to Gapdh mRNA values. (*: p<0.05, N.S.: not significant; A-D).

Mouse bone marrow cells were cultured with Myo-EVs or FFSS-Myo-EVs in the presence or absence of RANKL and M-CSF to compare the effects of FFSS to C2C12 cells on muscle cell-derived EV-suppressed osteoclast formation from mouse bone marrow cells. Treatment with both Myo-EVs or FFSS-Myo-EVs significantly suppressed the number of TRAP-positive MNCs, and FFSS-Myo-EVs more potently suppressed the number of TRAP-positive MNCs, compared to Myo-EVs (Fig 2A). Moreover, FFSS-Myo-EVs more potently suppressed RANKL-induced osteoclast-related gene mRNA levels, such as nuclear factor of activated T-cells cytoplasmic 1 (NFATc1) and dendritic cell-specific transmembrane protein (DC-STAMP), in mouse bone marrow cells, compared to Myo-EVs, and FFSS-Myo-EVs seemed to suppress RANKL-induced TRAP and cathepsin K (CTSK) mRNA levels more potently in mouse bone marrow cells without significant difference, compared to Myo-EVs (Fig 2B).

During osteoclast differentiation, mitochondrial content and metabolism increases, and oxygen consumption rate is high in mature osteoclasts [24]. We previously reported that Myo-EVs suppress mitochondrial biogenesis and oxygen consumption during osteoclast differentiation [16]. We therefore compared the suppressive effects of both Myo-EVs and FFSS-Myo-EVs on mitochondrial biogenesis during osteoclast differentiation with an Extracellular Flux Analyzer. Both Myo-EVs and FFSS-Myo-EVs significantly suppressed RANKL-induced OCR of mouse bone marrow cells, and FFSS-Myo-EVs seemed to suppress OCR more potently without significant difference, compared to Myo-EVs (Fig 2C). FFSS-Myo-EVs significantly suppressed RANKL-induced mitochondrial biogenesis-related gene mRNA levels, such as peroxisome proliferator-activated receptor gamma coactivator 1-β (PGC1β), a master regulator of mitochondrial biogenesis, nicotinamide adenine dinucleotide dehydrogenase subunit 4 (ND4) and cytochrome C (CytC), in mouse bone marrow cells, compared to Myo-EVs (Fig 2D).

**Fig 3:**
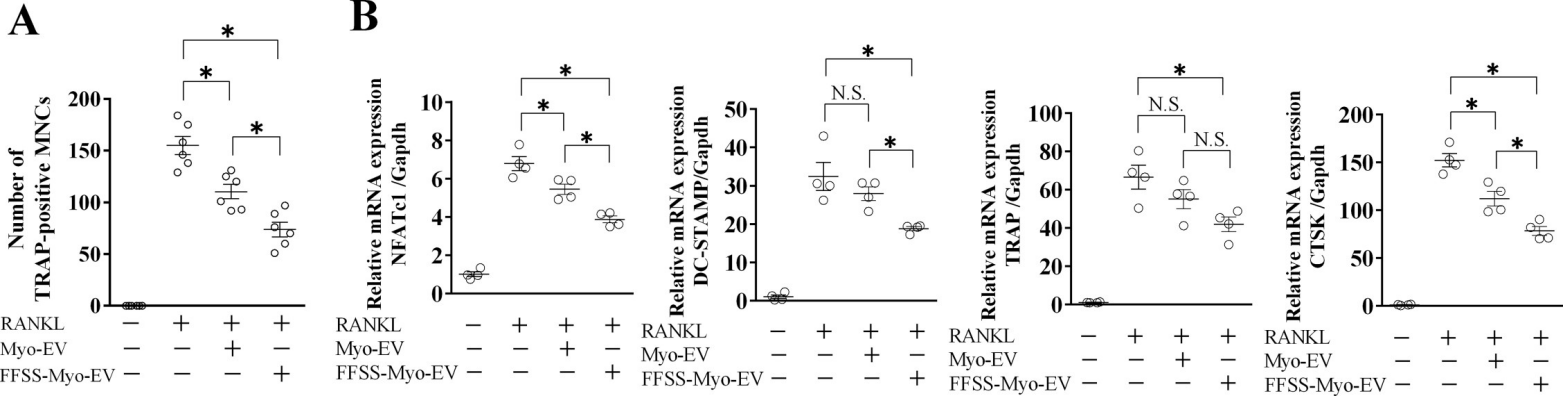
Effects of Myo-EVs secreted from C2C12 cells exposed with or without FFSS on osteoclast formation and mitochondrial biogenesis in Raw264.7 cells. (A) Raw264.7 cells were cultured with and without Myo-EVs or FFSS-Myo-EVs (4 μg/mL) in the presence or absence of RANKL (50 ng/mL) for 5 days. Numbers of TRAP-positive multinucleated cells (MNCs) in a 96-well plate were quantified. Data represent mean ± SEM of 6 experiments in each group. (B) Total RNA of Raw264.7 cells cultured with and without Myo-EVs or FFSS-Myo-EVs (4 μg/mL) in the presence or absence of RANKL (50 ng/mL) for 5 days was extracted for gene expression analysis of TRAP, NFATc1, DC-STAMP, CTSK or Gapdh by quantitative real-time PCR. Data represent mean ± SEM of 4 experiments in each group and are expressed relative to Gapdh mRNA values. (*: p<0.05, N.S.: not significant; A and B).

Since mouse bone marrow cells include various cells other than osteoclast precursors, we used mouse monocytic Raw264.7 cells to confirm the effects of FFSS to C2C12 cells on muscle cell-derived EV-suppressed osteoclast formation. Treatment with FFSS-Myo-EVs significantly suppressed the number of TRAP-positive MNCs in Raw264.7 cells (Fig 3A). Moreover, it significantly suppressed the mRNA levels of NFATc1, DC-STAMP and CTSK enhanced by RANKL, compared to treatment with Myo-EVs, although the effects of Myo-EVs on RANKL-induced TRAP mRNA levels were similar between FFSS-Myo-EVs and Myo-EVs (Fig 3B).

### Effects of FFSS on miRNA in Myo-EVs secreted from C2C12 cells

We speculated that miRNAs, which are abundant in muscle cells and Myo-EV and are less expressed in bone tissues, may play a pivotal role in the linkage from muscle to bone, when delivered from muscle to bone through EVs. We therefore performed small RNA-seq analysis to identify the miRNAs whose content in muscle cell-derived EVs is elevated by FFSS and is abundant compared to those in mouse primary osteoblasts and bone marrow cells. We extracted 18 miRNAs that normalized expression values were greater than 1000 in FFSS-Myo-EVs, and the values in FFSS-Myo-EVs were elevated at more than 1.25-fold, compared to Myo-EVs (Table 2).

**Table 2:** Normalized expression values of micro RNAs in Myo-EVs (EV), Myo-EVs secreted from C2C12 cells exposed with fluid flow shear stress (FFSS-EV), mouse primary bone marrow cells (BM) and mouse primary osteoblasts (OB).

	Myo-EV	FFSS-EV	Ratio of FFSS-EV/EV	BM	OB	Ratio of FFSS-EV/BM	Ratio of FFSS-EV/OB
mir-3473e	952	2584	2.71	216	17	11.98	155.65
mir-6236	664	1179	1.77	103	56	11.50	20.94
mir-155	855	1512	1.77	369	249	4.10	6.07
mir-26a	7039	12160	1.73	22269	19415	0.55	0.63
mir-224	2521	4198	1.67	89	664	47.17	6.32
mir-23a	1838	2894	1.57	16970	7538	0.17	0.38
mir-221	2954	4575	1.55	26925	4688	0.17	0.98
mir-7a	3919	5968	1.52	11779	5130	0.51	1.16
mir-690	891	1341	1.51	85	60	15.85	22.27
mir-199a	8731	13026	1.49	1408	66862	9.25	0.19
mir-196a	7813	10952	1.40	119	2	92.04	5476.20
mir-871	2572	3483	1.35	0	0	−	−
mir-99a	4729	6371	1.35	13235	35768	0.48	0.18
mir-222	1600	2138	1.34	16912	1941	0.13	1.10
mir-100	4861	6406	1.32	1598	56852	4.01	0.11
mir-27b	20006	25674	1.28	129665	147286	0.20	0.17
mir-98	5429	6822	1.26	4546	3449	1.50	1.98
mir-152	7129	8895	1.25	2688	41176	3.31	0.22

**Fig 4:**
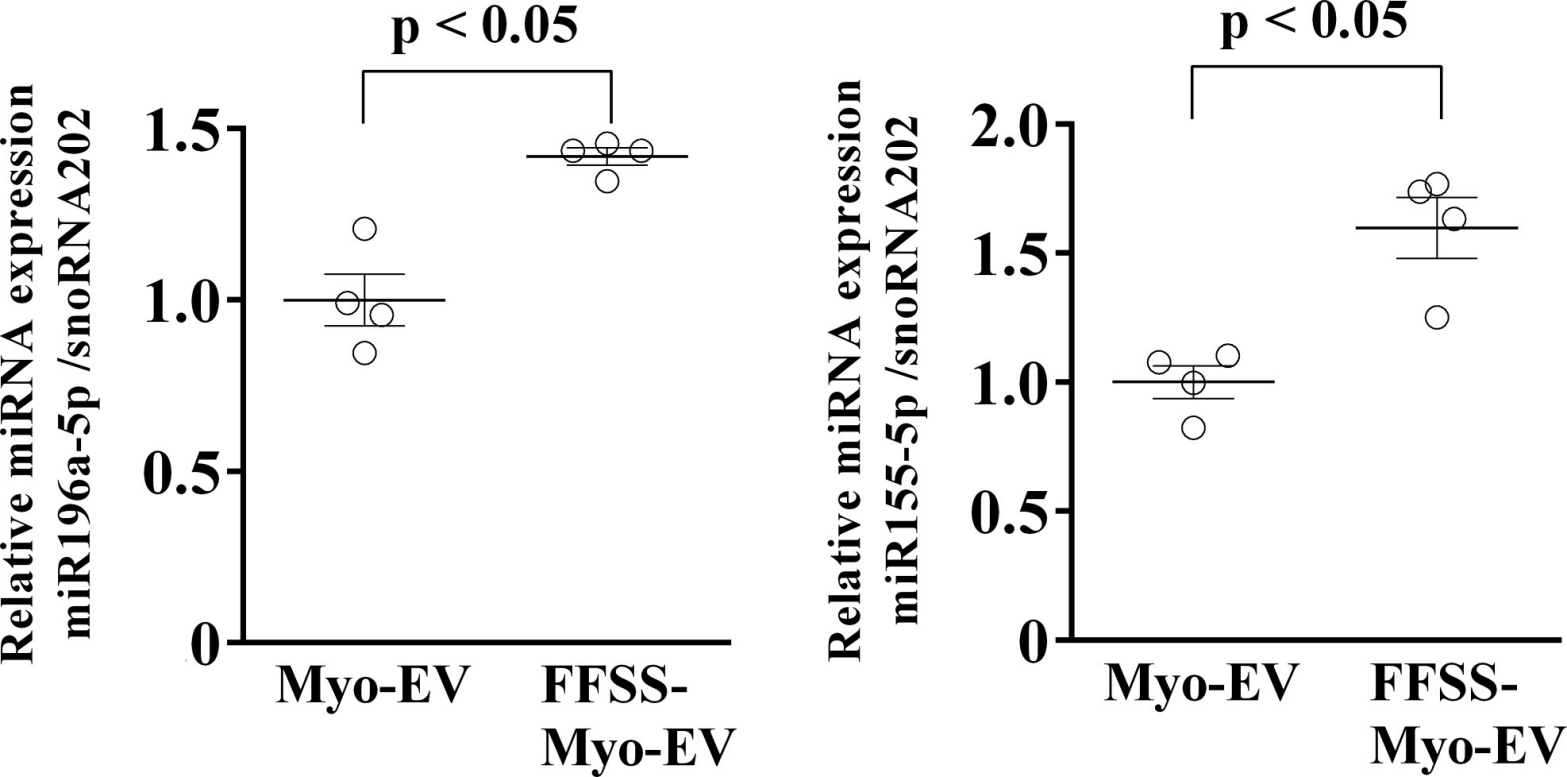
Expression of miRNA 155-5p and miRNA 196a-5p in Myo-EVs and Myo-EVs secreted from C2C12 cells exposed with FFSS (FFSS-Myo-EVs). Total RNA from both Myo-EVs and FFSS-Myo-EVs was extracted for the analysis of miRNA expression by quantitative real-time PCR. Data represent mean ± SEM of 4 experiments in each group and are expressed relative to miRNA values of snoRNA202.

Among the extracted miRNAs, miR155 and miR196a were expressed at low level in mouse primary osteoblasts and bone marrow cells (with high ratio of FFSS-EV/ BM and ratio of FFSS-EV/ OB in Table 2) and have been previously reported to be involved in bone metabolism, including the suppressive activity of osteoclast formation [15,25–27]. We then examined the effects of FFSS on the contents of miR196a-5p and miR155-5p in Myo-EVs. FFSS significantly elevated miR196a-5p and miR155-5p levels in EVs from C2C12 cells in quantitative real-time PCR (Fig 4).

### Effects of FFSS-Myo-EVs on the phenotypes of mouse primary osteoblasts

**Fig 5:**
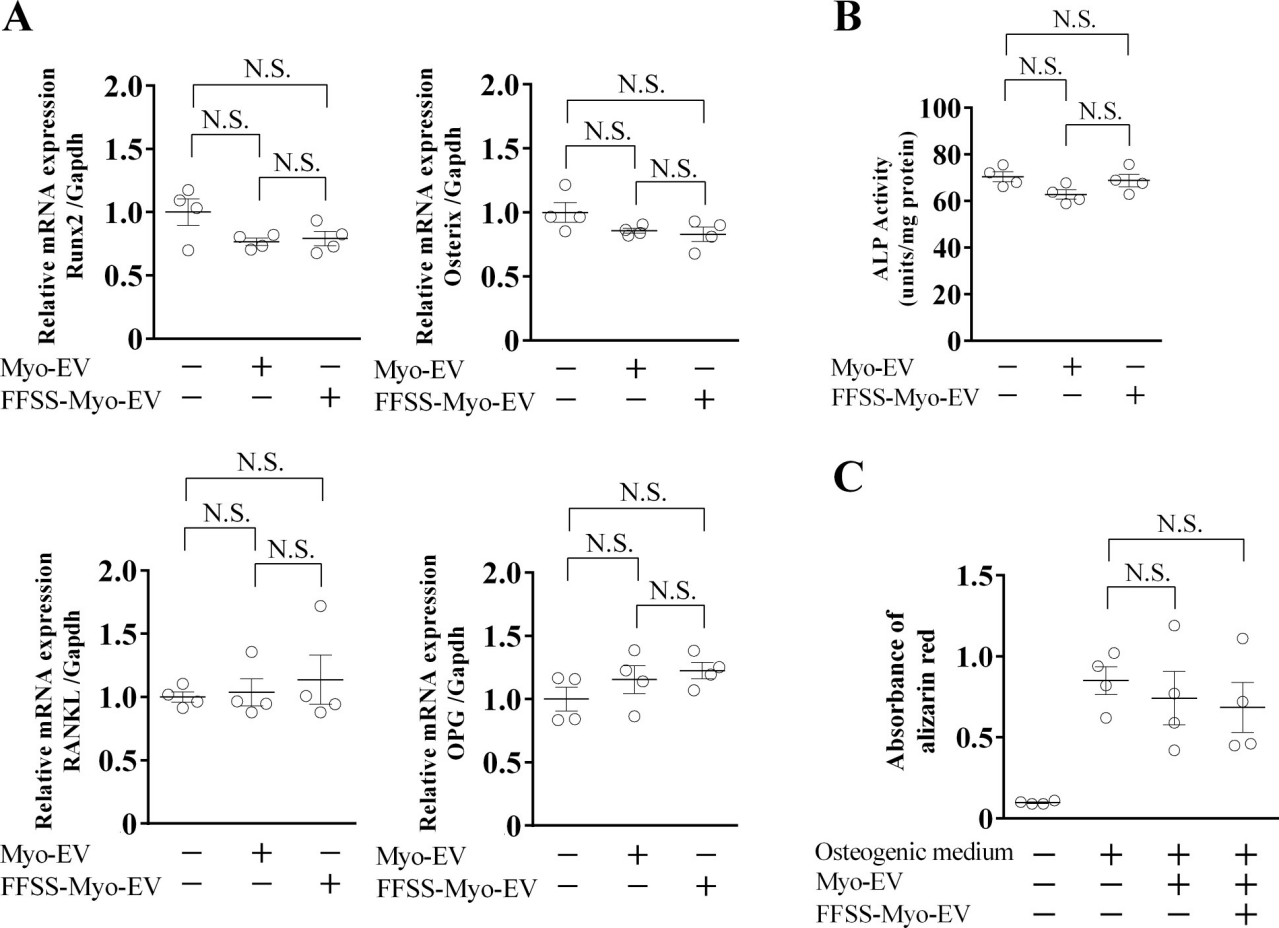
Effects of Myo-EVs secreted from C2C12 cells exposed with or without FFSS on mouse osteoblasts. (A) Mouse osteoblasts were cultured with and without Myo-EVs or FFSS-Myo-EVs (4 μg/mL) for 24 hr. Total RNA of cells was extracted for gene expression analysis of Runx2, Osterix, RANKL, OPG or Gapdh by quantitative real-time PCR. Data are expressed relative to Gapdh mRNA values. (B) Mouse osteoblasts were cultured with and without Myo-EVs or FFSS-Myo-EVs (4 μg/mL) for 48 hr. Then, ALP activity of the cells was measured. (C) Primary osteoblasts were cultured with and without Myo-EVs or FFSS-Myo-EVs (4 μg/mL) in normal medium or osteogenic medium containing 10 mM β-glycerophosphate and 50 μg/mL ascorbic acid for 2 weeks. Then, the cells were fixed and stained with Alizarin red. Absorbance at 550 nm of the extracted stain from cells was measured. Data represent mean ± SEM of 4 experiments in each group (A-C). N.S.: not significant.

Mouse primary osteoblasts were cultured in the presence or absence of Myo-EVs or FFSS-Myo-EVs to examine the effects of FFSS to C2C12 cells on the osteoblast phenotypes. Both Myo-EVs and FFSS-Myo-EVs did not affect the mRNA levels of osteogenic genes [Runt-related transcription factor-2 (Runx2) and Osterix] and bone resorption-related genes [RANKL and osteoprotegerin (OPG)] (Fig 5A) as well as ALP activity (Fig 5B) and mineralization (Fig 5C) in mouse primary osteoblasts.

## Discussion

Mechanical stress is important for muscle and bone homeostasis, and mechanical unloading induces muscle atrophy and bone loss in humans and animals [3]. FFSS is included in a biologically important mechanical stress in skeletal muscles, and it affects the levels of myogenic factors and signaling in muscle cells [4,28]. In the present study, FFSS-Myo-EVs more potently suppressed osteoclast formation and the expression of osteoclast-related genes (NFATc1 and DC-STAMP) and mitochondrial biogenesis markers (PGC1β, ND4 and CytC) enhanced by RANKL than Myo-EVs in mouse bone marrow cells. Moreover, FFSS-Myo-EVs more potently suppressed osteoclast formation and the expression of osteoclast-related genes (NFATc1, DC-STAMP and CTSK) enhanced by RANKL than Myo-EVs in Raw264.7 cells. These data indicate that FFSS influences EVs secreted from mouse muscle cells, then leading to an enhancement of muscle cell-derived EV-induced suppressive effects on osteoclast formation and mitochondrial biogenesis in mouse cells. A previous study indicated that mechanical stress enhances the production of EVs in osteocytes through mechanical stress-induced calcium oscillations in mice, which might be related to mechanical stress-induced bone formation [17]. Recently, various mechanoresponsive miRNAs were identified, and these miRNAs have been studied as novel biological regulators in muscle and bone [29]. miR-132 induced by FFSS regulates the differentiation and proliferation in human periodontal ligament cells [30], and mechanical stress-responsive miR-29b-3p in mouse osteocytes regulates IGF-1 secretion and osteoblast differentiation in mice [31]. In addition, Hua et al. reported that the miRNA expression profiles in C2C12 cells are regulated by mechanical stress via NF-κB activation [19]. These findings suggest that mechanical stress might regulate miRNAs contained in EVs, leading to a modulation of the physiological effects of EVs in the muscle/bone interactions. Since FFSS did not affect protein amount and particle size of muscle cell-derived EVs in the present study, we can speculate that FFSS might modulate some factors, including miRNAs, in muscle cell-derived EVs. Since the enhanced effects of FFSS-Myo-EVs on mitochondrial biogenesis seemed to be less potent, compared to those of FFSS-Myo-EVs on osteoclast formation from mouse bone marrow cells in our study, FFSS-responsive factors contained in muscle cell derived-EVs might partly affect osteoclast formation through the mechanisms other than the mitochondrial function in mice.

Small RNA-seq analysis revealed that the expression of some miRNAs in muscle cell-derived EVs were elevated by applying FFSS to C2C12 cells in the present study. Among the miRNAs, miR155-5p and miR196a-5p levels in muscle cell-derived EVs were significantly elevated by FFSS and were extremely low in primary mouse osteoblasts and bone marrow cells. We recently reported that transfection of miRNA mimic of miR155-5p and miR196a-5p suppressed osteoclast-like cell formation and transfection of miRNA mimic of miR196a-5p suppressed the expression of osteoclast-related genes (NFATc1, TRAP and CTSK), the OCR and mitochondrial biogenesis markers (PGC1β and ND4) enhanced by RANKL in Raw264.7 cells [15]. In addition, it was reported that miR155 attenuates osteoclast formation by targeting SOCS1 and MITF, essential regulators of osteoclast formation in mouse cells [27]. These findings suggest that miR155-5p and miR196a-5p might be responsible for an enhancement of FFSS to C2C12 cells on muscle cell-derived EV-suppressed osteoclast formation in mouse cells. It was reported that miR-196a regulates osteogenic differentiation of human adipose-tissue derived stem cells (hASCs), which might be mediated by HOXC8 [26]. Moreover, Ai et al revealed that miR196a inhibits adipogenic differentiation and promotes osteogenic differentiation in hASCs by regulating Wnt-β-catenin pathway [25]. In the present study, both Myo-EVs and FFSS-Myo-EVs did not affect the expression of osteoblast-related genes (Runx2, Osterix, RANKL and OPG), ALP activity and mineralization in mouse primary osteoblasts. Although the reason of these discrepancy has still remained unknown, the other miRNA in muscle cell-derived EVs might modulate the effects of miR-196a-5p on the osteoblast phenotypes. Further studies will be necessary to clarify the roles of miR196a-5p in muscle cell-derived EVs on the osteoblast phenotypes.

FFSS elevated the expression of miR26a and miR100 in Myo-EVs in our small RNA-seq analysis. miR100-5p prevents osteoclast formation and bone loss in ovariectomy-induced osteoporotic mice by targeting FGF-21 [32]. miR26a is up-regulated during osteoclastogenesis, then inhibiting RANKL-induced osteoclast formation [33]. These findings suggest that several miRNAs, such as miR100 and miR26a, in muscle cell-derived EVs might be involved in an enhancement of FFSS to C2C12 cells on muscle cell-derived EV-suppressed osteoclast formation in mouse cells. However, since the expression of miR-26a and miR100 in mouse primary osteoblasts or bone marrow cells were higher, compared to miR155-5p and miR-196a, the physiological significance of miR26a and miR100 in muscle cell-derived EVs in the muscle/bone interactions might be minor.

There are several limitations in the present study. First, we could not clarify whether the changes in the content of miR196a-5p and miR155-5p in Myo-EVs directly affect the activity of FFSS-Myo-EV on osteoclast formation in this study. Since various miRNAs are included in EVs, we cannot rule out the possibility that other miRNAs might be involved in an enhancement of osteoclast formation suppressed by Myo-EVs by FFSS to C2C12 cells. Further studies will be necessary to clarify the pivotal miRNA in muscle-bone interactions through Myo-EVs. Second, the properties of EVs secreted from C2C12 cells and Raw264.7 cells might be partly different those of EVs from muscle tissues and osteoclast precursors, respectively. We therefore cannot rule out the possibility that this *in vitro* model system used in the present study might not represent the linkage of muscle to bone through EVs *in vivo*.

In conclusion, we first showed that FFSS to C2C12 cells enhances muscle cell-derived EV-suppressed osteoclast formation and mitochondrial biogenesis in mouse cells. Moreover, miR155-5p and miR196a-5p might be related to the modulation of muscle cell-derived EVs by FFSS in C2C12 cells.

## Abbreviation

*CTSK*: Cathepsin K
*CytC*: Cytochrome C
*DC-STAMP*: Dendritic cell-specific transmembrane protein *Gapdh*: Glyceraldehyde-3-phosphate dehydrogenase
*ND4*: Nicotinamide adenine dinucleotide dehydrogenase subunit 4
*NFATc1*: Nuclear factor of activated T-cells cytoplasmic 1
*OPG*: Osteoprotegerin
*PGC1-β*: Peroxisome proliferator-activated receptor gamma coactivator 1-beta
*RANKL*: Receptor activator of NF-κB ligand
*Runx2*: Runt-related transcription factor 2
*TRAP*: Tartrate resistant acid phosphatase
